# The relevance of reducing Veress needle overshooting

**DOI:** 10.1038/s41598-023-44890-1

**Published:** 2023-10-14

**Authors:** T. Horeman-Franse, R. R. Postema, T. Fischer, J. Calleja-Agius, C. Camenzuli, L. Alvino, S. F. Hardon, H. J. Bonjer

**Affiliations:** 1 Department of Biomechanical Engineering, Delft University of Technology, TU-Delft, Mekelweg 2, 2628CD Delft, The Netherlands; 2 Department of Surgery, Amsterdam University Medical Centers, Location VUMC, Amsterdam, The Netherlands; 3 Department of Anatomy, Faculty of Medicine and Surgery, University of Malta, Msida, Malta; 4 Neyenrode Business School, Amsterdam, The Netherlands; 5 European Association of Endoscopic Surgery, Eindhoven, The Netherlands

**Keywords:** Risk factors, Medical research

## Abstract

Safe insertion of the Veress needle during laparoscopy relies on the surgeons’ technical skills in order to stop needle insertion just in time to prevent overshooting in the underlying organs. To reduce this risk, a wide variety of Veress needle systems were developed with safety mechanisms that limit the insertion speed, insertion depth or decouple the driving force generated by the surgeon’s hand on the needle*.* The aim of this study is to evaluate current surgeons’ perceptions related to the use of Veress needles and to investigate the relevance of preventing overshooting of Veress needles among members of the European Association of Endoscopic Surgery (EAES). An online survey was distributed by the EAES Executive Office to all active members. The survey consisted of demographic data and 14 questions regarding the use of the Veress needle, the training conducted prior to usage, and the need for any improvement. A total of 365 members residing in 58 different countries responded the survey. Of the responding surgeons, 36% prefer the open method for patients with normal body mass index (BMI), and 22% for patients with high BMI. Of the surgeons using Veress needle, 68% indicated that the reduction of overshoot is beneficial in normal BMI patients, whereas 78% indicated that this is beneficial in high BMI patients. On average, the members using the Veress needle had used it for 1448 (SD 3031) times and felt comfortable on using it after 22,9 (SD 78,9) times. The average years of experience was 17,6 (SD 11,1) and the surgeons think that a maximum overshoot of 9.4 (SD 5.5) mm is acceptable before they can safely use the Veress needle. This survey indicates that despite the risks, Veress needles are still being used by the majority of the laparoscopic surgeons who responded. In addition, the surgeons responded that they were interested in using a Veress needle with an extra safety mechanism if it limits the risk of overshooting into the underlying structures.

Safe establishment of a pneumoperitoneum is a key step at the beginning of every laparoscopic procedure. Although several methods are being used, there is still no consensus on how to penetrate the abdominal wall for insufflation^1^. Since the late 1980s, the use of Veress needles became the standard of care for several laparoscopic procedures^2,3^. One of the advantages of the Veress needle when compared to other techniques such as the open or Hasson approach, is that it takes less time to insert the trocar with less scarring, due to a smaller incision. This became more relevant with the increase in patients with higher mean body mass index (BMI) who often require a larger incision^4^. However, this closed entry approach using the Veress needle relies on the surgeons’ technical skill in order to stop needle insertion just in time to prevent overshooting in the underlying organs, such as the intestines or even major abdominal arteries and veins.

To prevent the problem of overshooting, suggestions were made in the literature to add mechanisms to fixate the needle or inner stylet as soon as it shoots in position^5^, or by expanding the blunt area of the tip directly after insertion when the tip hits the underlying organs or structures^6,7^. Other studies suggested to add monitoring systems based on utilizing acoustic emissions or optic information recorded at the tip of the needle^8,9^. Lastly, research groups added vacuum cups around the needle shaft to remove air above the abdominal wall in order to lift it during puncturing^10^ or even added robotic arms to prevent acceleration of the needle when entering the abdominal wall^11^. Although literature indicates that all these approaches can limit overshooting in theory, their impact on the surgical workflow and dependency on the infrastructure and facilities within the operating room remains difficult to understand due to the absence of implementation studies.

In an attempt to find an affordable solution that stays close to the original design and that does not rely on additional measurement devices, actuators, or vacuum systems, the veressPLUS safety mechanism has been developed recently^12^. This safety mechanism decouples the driving force generated by the hand and arm of the user as soon as the needle punctures through the peritoneum and was first validated in a benchtop model and subsequently in a pre-clinical setting on Thiel embalmed bodies at the Department of Anatomy at the University of Malta^12,13^. Both studies indicate that the mechanism functions well, as it significantly reduces overshooting independently from the users’ experience level and gender. During the use of the prototypes, user feedback indicated that different needle grips are being used by surgeons. Moreover, some surgeons even suggested that inexperienced Veress needle users jeopardize patient safety by holding the Veress needle in a wrong way, while others opposed this by claiming that, with practice, there is no evidence that there is an increase in safety upon first entry of the abdominal cavity. Finally, some surgeons suggested to use alternative locations on the body for insertion, e.g. left subchondral region, that were not included in the study. Therefore, the relevance and the best approach to integrate it in a standard Veress needle design is not fully understood yet, despite the good results. In an attempt to define user requirements better, literature about the insertion procedure was reviewed. Studies by Vaishnani et al. and Datey et al. indicate that there are no significantly different outcomes when open and closed techniques are compared in a more generic way^14,15^. However, Azevedo et al., reported that insertion at the midline should be avoided as the risks related to overshooting in organs is too high^16^. Moreover, the 2021 guideline of the Society of Obstetricians and Gynecologists of Canada states that, although conventionally the most common site to insert the Veress needle is the umbilical area, there are a lot of reasons why this area should be avoided. These reasons are again underlining the risk of accidental penetration of the needle in the underlying organs due to for example, previous surgery, clinical history or anatomical and BMI variations^17^.

As this kind of generic Veress needle related feedback and procedural insights have influence on the final appearance, feel and function of a Veress needle with integrated VeressPLUS safety mechanism, it became evident that qualitative user feedback is needed before the final design can be made. Therefore, the aim of this study was to identify and evaluate current perceptions related to the use of Veress needles in daily practice and to investigate the relevance of preventing overshooting of the needles among European Association of Endoscopic Surgery (EAES) members. Furthermore, the type of training which has been conducted prior to using the Veress needle during laparoscopic surgery, was determined.

## Methods

An online survey was conducted using a questionnaire designed by the EAES Technology Committee and distributed by the Executive Office to all 3621 active members of the EAES, and a reminder was sent three weeks later. The survey was conducted between January 4th and March 4th, 2022. It consisted of 31 questions regarding the Veress needle and its use, as presented in Table 1.

**Table 1:** Survey questions.

Q1. What is your preferred method for establishing a pneumoperitoneum in patients with a normal Body Mass Index (BMI)?
Q2. What is your preferred method for establishing a pneumoperitoneum in patients with a high BMI?
Q3. Roughly, after how many procedures were you comfortable enough in using the Veress needle?
Q4. Roughly, after how many procedures were you comfortable enough in using the Hasson method?
Q5. When did you pick your preferred method? *During training/*Following personal experience during surgery/Other
Q6. Where did you learn how to perform a pneumoperitoneum? In the clinical skills lab/on the job training/Other
Q7. What is your preferred (primary) entry location in normal BMI patients? Lee-Huang point/Palmer’s point/Umbilical point
Q8. What is your preferred (primary) entry location in high BMI patients? Lee-Huang point/Palmer’s point/Umbilical point
Q9. How do you hold the Veress needle (see images below)? At the shaft/At the base
Q10. Do you have a preferred type of Veress needle? Yes/No
Q11. If yes, which one and why do you prefer it?
Q12. Do you agree with the following statement: Decreasing overshooting increases the overall safety of the Closed technique no 1/2/3/4/5 yes
Q13. Do you agree with the following statement: Decreasing the learning curve of the Veress Needle use increases the overall safety of the Closed technique no 1/2/3/4/5 yes
Q14. Do you agree with the following statement: Increase in Body Mass Index is directly related to an increased risk of complications during first entry no 1/2/3/4/5 yes
Q15. Do you agree with the following statements: Improving the sustainability of hospital processes and operations is important to me no 1/2/3/4/5 yes
Q16. In your opinion, how far beyond the peritoneum (Ideal Insertion Depth) does the Veress needle tip need to be for safe insufflation (in mm)?
Q17. In adopting new surgical technology/products do you consider yourself an: Innovator/Early adopter/Early majority/Late majority/Laggard
Q18. What are your main concerns about using NEW medtech devices during surgery?
Q19. If the above claims are true would you consider using the new VeressPLUS in patients with a normal BMI? no 1/2/3/4/5 yes
Q20. Please explain your answer
Q21. If the above claims are true, would you consider using the new VeressPLUS in patients with a high BMI?
Q22. Please explain your answer
Q23. Age
Q24. Gender
Q25. Nationality
Q26. Years of experience (as a surgeon)
Q27. Estimated number of Veress needle use
Q28. Place of work Training hospital/Non-training hospital/Private clinic/Academia/Other
Q29. Surgical specialty
Q30. Dominant hand
Q31. Would you be interested in joining the VeressPLUS R&D project as an innovator/early adopter?
If yes, please share your contact details below

Furthermore, the questions were designed to determine the participants’ opinions on disposable/reusable instruments, device performance, safety and training and gathering background information of the participants. Frequency distribution was used to analyze the data (method name, supplier, company, country, version). The questions in their original form with illustrations can be found in supplemental file 1. The EAES Research Committee decided that this inventory study based on an online questionnaire did not require IRB approval. All procedures were performed in accordance with relevant guidelines in the manuscript and according to the Helsinki declaration. The informed consent form that was used in the introduction email to the EAES members can be found in supplemental file 2. All the participants provided written informed consent. All data generated or analysed during this study are included in the supplemental files of this published article.

### Correlation

To gain more insight in possible relations between data, Pearson correlation was conducted between all numerical questions and presented as a correlation matrix. In the Pearson correlation matrices (SPSS 17), the following guidelines are being proposed for the strength of correlation. A small correlation gives absolute values between 0.1 and 0.3. A medium correlation gives absolute values between 0.3 and 0.5. A large correlation gives absolute values between 0.5 and 1^18^.

## Results

A total of 365 members (out of 3621) EAES members of whom all were, or used to be, practicing surgeons, responded. All anonymized data can be found in supplemental file 3. What follows are the highlights of the data.

### General questions

**Figure 1:**
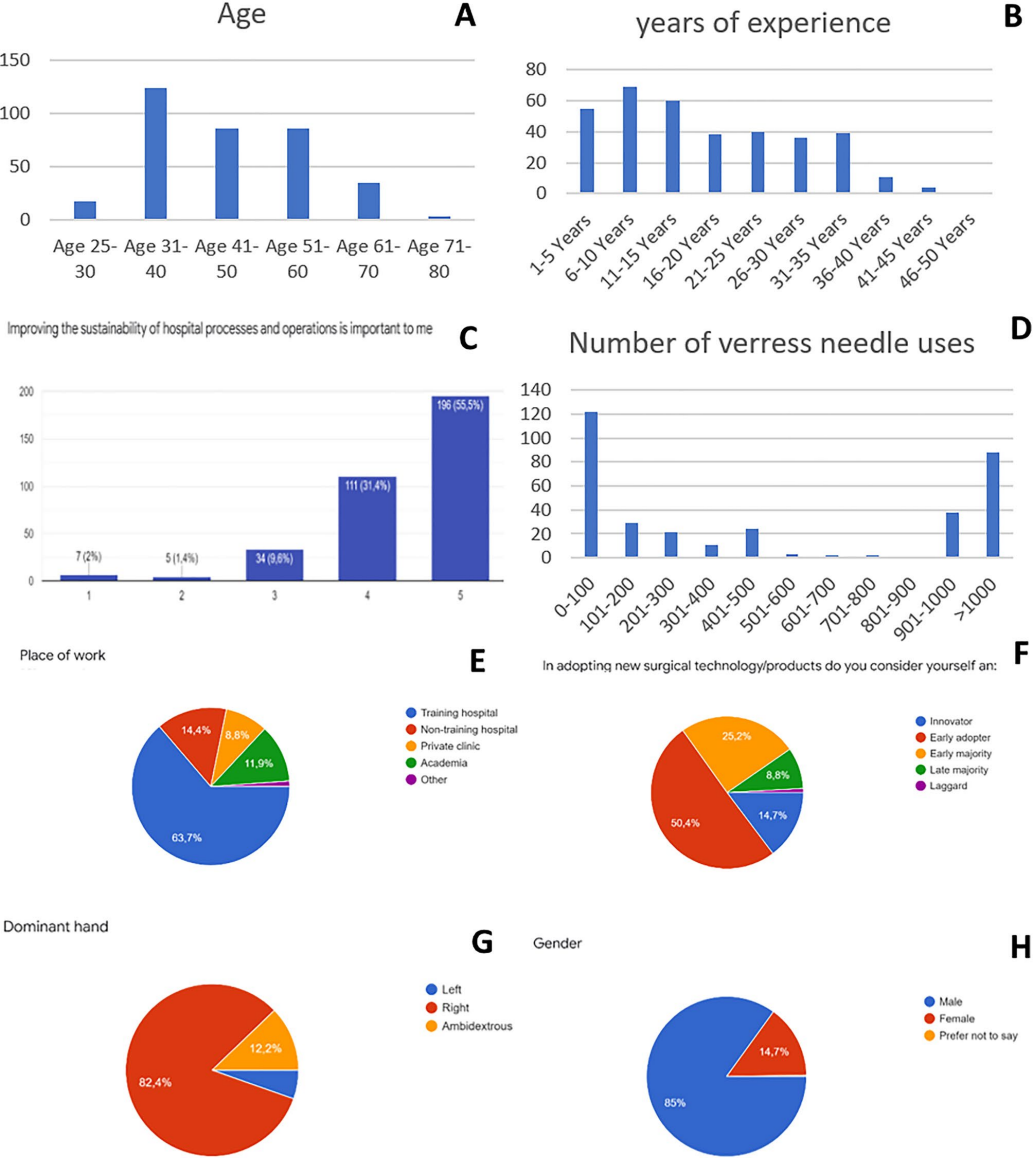
Survey data for the general questions. (**A**) Age, (**B**) years of experience, (**C**) importance of sustainability, (**D**) number of times the Veress needle was used, (**E**) place of work, (**F**) how do you consider yourself when adopting new technology, (**G**) dominant hand, (**H**) gender.

The 85% male and 15% female respondents originated from 58 countries with the highest proportion from Italy (75), the Netherlands (23), and Romania (21). Regarding the age of the respondents, the largest group was found in the range of 31–40 years (124) followed by the range of 41–50 and 52–60 years among 86 of the respondents (Fig. 1A). Most of the respondents had 6–10 years of experience followed by 11–15 years and 1–5 years of experience (Fig. 1B). When asked if sustainable hospital processes are important for the respondent, the majority agreed (307), of whom 196 strongly agreed (Fig. 1C). The vast majority of the 365 respondents claimed to use the Veress needle between 0 and 100 times (122) or more than 1000 times (88) (Fig. 1D). The remaining 155 respondents were divided over the rest of the group in much lower numbers. In total, 63.7% of the surgeons worked in a training hospital, 14.4% in a non-training hospital and 8.8% in a private clinic (Fig. 1E). From all respondents, 85.4% consider themselves as early adapter, 25.2% as early majority and 14.7% as innovator (Fig. 1F). In addition, 82.4% of all respondents were right-handed and interestingly, more respondents were ambidextrous (12.2%) than left handed (5.4%) (Fig. 1G). From all respondents, 85% was male and 14.7% female (Fig. 1H).

### Procedural questions

**Figure 2:**
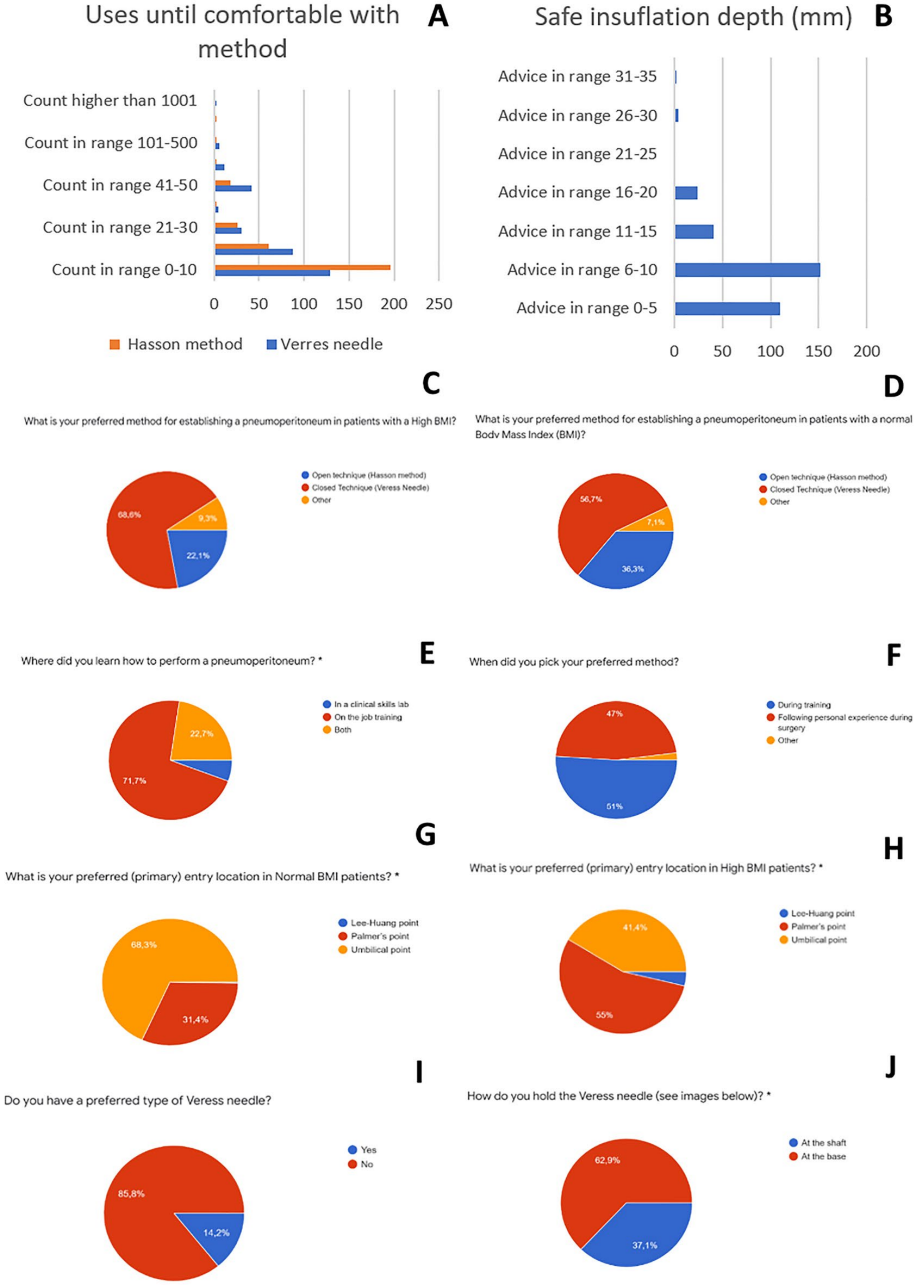
Survey data for the procedural questions. (**A**) number of attempts before feeling comfortable with the Veress, (**B**) considered safe insufflation depth, (**C**) preferred method of insufflation for patients with high BMI, (**D**) preferred method of insufflation for patients with normal BMI, (**E**) where did you learn to perform a pneumoperitoneum, (**F**) when did you pick your preferred method of insufflation, (**G**) preferred location of insufflation for patients with high BMI, (**H**) preferred location of insufflation for patients with normal BMI, (**I**) do you have a preferred type of Veress needle, (**J**) how do you hold your Veress needle.

Independent from the type of approach, more than 50% of the respondents suggest that up to 20 trials are needed before the Veress needle technique is mastered (Fig. 2A). The respondents think that a maximum overshoot of the Veress needle in the abdomen of 9.4 (SD5.5) mm is acceptable in order to use the system safely (Fig. 2B). There is a difference between respondents on how they handle obese patients during insufflation and preparation of the location of insertion.

**Figure 3:**
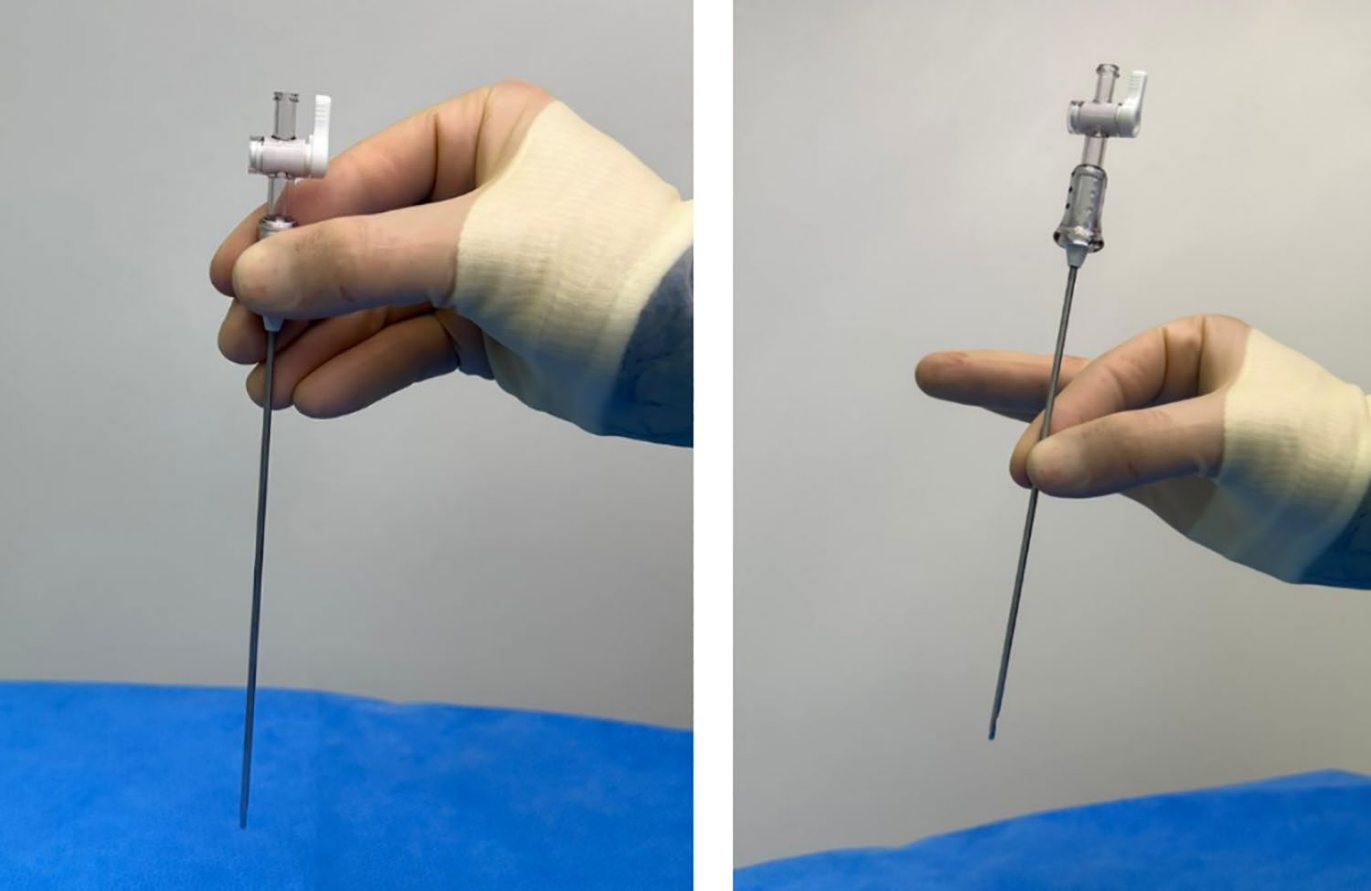
Survey question regarding the grip of the hand on the standard Veress needle. Left, fingers on the Grip. Right, Fingers on the needle.

Out of the responding surgeons, 69% reported that the closed Veress technique is preferred over the open technique for high BMI, and 57% for normal BMI patients (Fig. 2C,D). The same data also showed that only 36% of the respondents think it is better to use the open method for normal patients, and 22% for high BMI. From the respondents, 72% were trained to use the Veress needle on the job, 5% in a skills lab and 23% from both (Fig. 2E). In this study, 51% indicated that they chose their preferred first access method after training, while 47% decided what worked best for them during practice (Fig. 2F). For patients with a normal BMI (15–25), the insertion area of choice was around the umbilicus for the majority of the respondents (68%) while for patients with a BMI higher than 25, this was the Palmer’s point (55%) (Fig. 2G,H). Eight-six percent confirmed that they have a preferred type of Veress needle (Fig. 2I). When asked if respondents have a preferred grip area (Fig. 2J), 37% answered that this is the shaft, while 63% reported that they hold the Veress needle at its grip area during insertion (Fig. 3).

**Figure 4:**
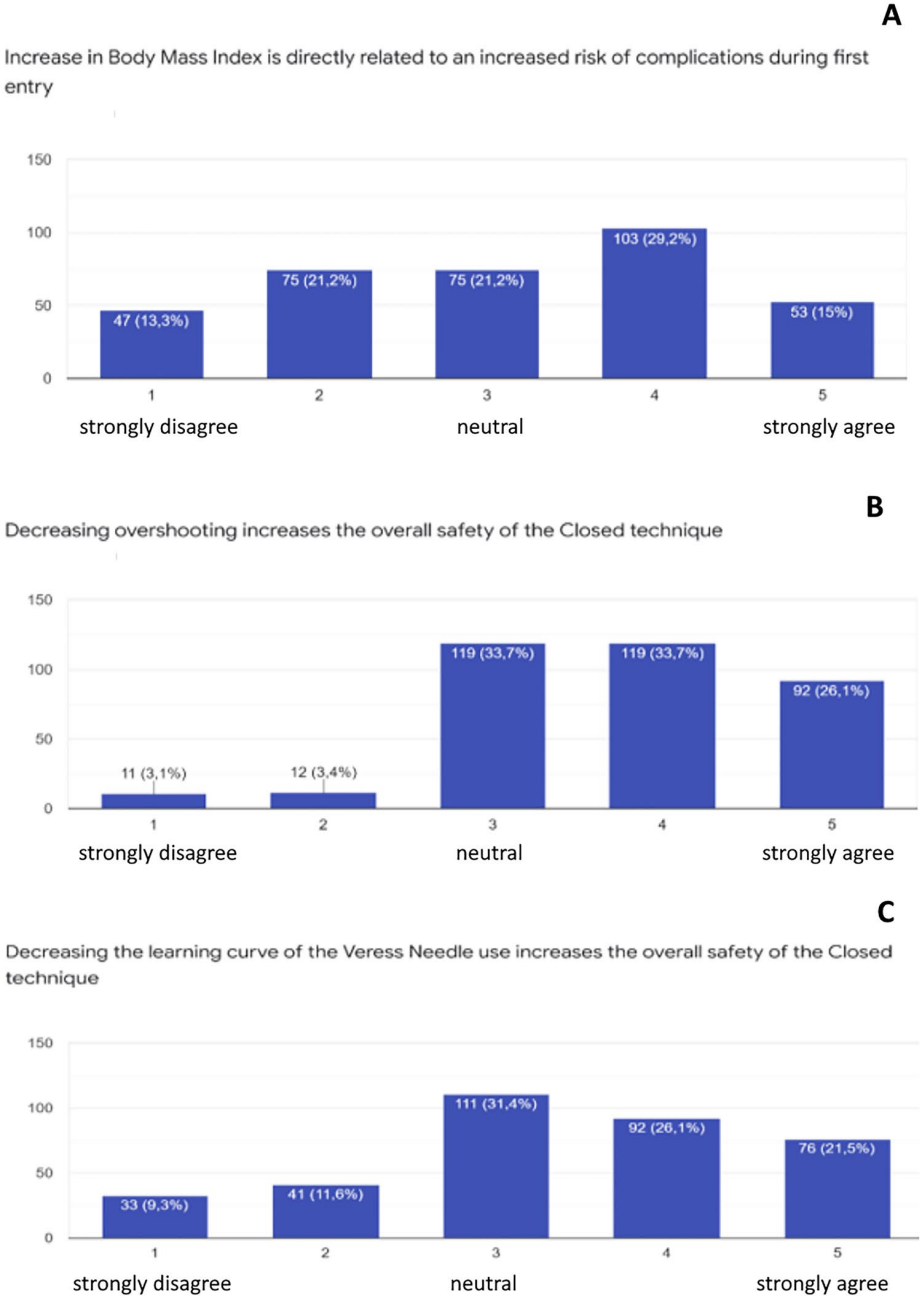
Survey data for the procedural questions. (**A**) increase in BMI is directly related to first entry complications, (**B**) decreasing overshooting increases the overall safety of the closed technique, (**C**) decreasing the learning curve of the Veress needle use increases the overall safety of the closed technique.

Figure 4A indicates that around 43% of the respondents confirmed that a high BMI is directly related to risk of complications, while 34% suggested that this is not the case. The rest of the respondents (21%) were neutral. When asked if a reduction in overshooting can reduce the risk of complications, 87% agreed, of whom 56% strongly agreed, 34% answered neutral and only 7% answered that this is not the case (Fig. 4B). When asked if a reduction in a learning curve for a new technology can decrease the risk of complications, 37% agreed, 42% answered neutral and 21% stated that this is not the case (Fig. 4C).

### VeressPLUS questions

**Figure 5:**
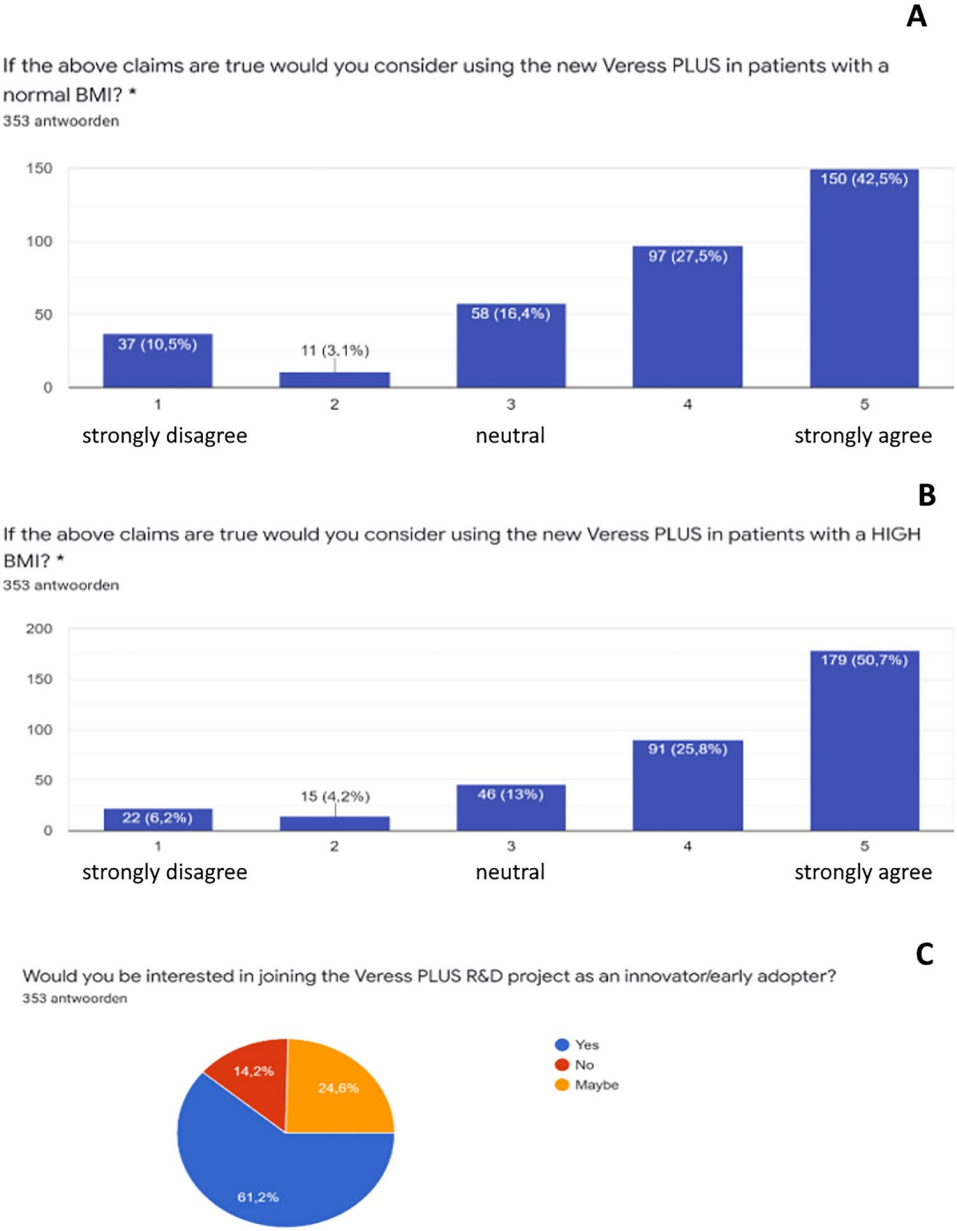
Survey data for the VeressPLUS innovation related questions. (**A**) If the above claims are true, would you consider using the new VeressPLUS in patients with a normal BMI? (**B**) If the above claims are true, would you consider using the new VeressPLUS in patients with a high BMI? (**C**) Would you be interested in joining the VeressPLUS project as an innovator/early adapter?

When asked if the respondents think a Veress needle that reduces overshoot such as the proposed prototype with integrated VeressPLUS safety mechanism^5^, should be used on patients with normal BMI, 70% agreed, of whom 43% strongly agreed, 16% answered neutral and 14% did not agree (Fig. 5A). When asked if the respondents thought a Veress needle that reduces overshoot should be used on patients with a high BMI, 76% agreed, of which 51% strongly agreed, 13% answered neutral and 10% disagreed (Fig. 5B). The last item of the survey enquired whether the respondents wished to join the VeressPLUS R&D collaborative project as an innovator or early adaptor. From the respondents, 61% agreed, 25% answered “maybe” while 14% was not interested at all (Fig. 5C).

### Correlation

Table 2 and supplemental file 2 indicate that only the question outcomes for “Age” and “years of experience” of the questionnaire were strongly related with a correlation coefficient of 0.94. In addition, the question outcomes for “If the above claims are true would you consider using a new needle with VeressPLUS safety mechanism in patients with a normal BMI” and “If the above claims are true would you consider using this new needle with VeressPLUS safety mechanism in patients with a normal BMI” were strongly correlated with a correlation coefficient of 0.66. The questions “years of experience” and “estimated number of Veress needle use” showed a medium correlation coefficient of 0.35 while the questions “Age” and “estimated number of Veress needle use” showed a medium correlation coefficient of 0.34. Another medium correlation of 0.33 was found between the question outcome “decrease in overshooting increases the overall safety” and “decreasing the learning curve of Veress needle increases the overall safety of closed entry techniques”. The rest of the questions were not or were only weakly related.

**Table 2:**
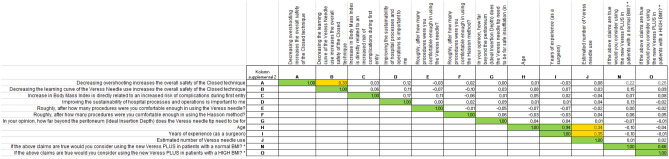
Pearson correlation matrix showing the medium and strong correlations indicated in orange and green.

## Discussion

This study, conducted using a questionnaire among 3621 EAES members, provides important insights and perceptions regarding the first access methods for safe establishment of the pneumoperitoneum in laparoscopic surgery, and more specifically, the use of the Veress needle. As more than 10% of active EAES members responded from all parts of the world, we are convinced that the results provide good insights into current practice of use of the Veress needle in laparoscopic (gastrointestinal, HPB, bariatric, oncologic, urologic, and gynecologic) surgery. However, it should be taken into consideration that more surgeons who use the Veress in daily practice or, have a strong opinion against using the Veress needle, answered the survey compared to surgeons who normally only practice the “open” approach. Therefore there is the possibility of bias, as the data may not reflect the use of Veress needles in practice with high accuracy.

The majority of the respondents were using the Veress needle in its current form. It is important to realize that most surgeons choose their preferred method during training (51%) and get familiar while training on the job (71%). This may indicate the need for training facilities with adequate realism for training to ensure that surgical residents and surgeons are skilled enough before starting to use the Veress needles on patients. Especially for patients with a higher BMI, it seemed that around 8% (category 5 grows from 43 to 51%) of the respondents become absolutely certain to use the Veress needle while they are usually less convinced. This is supported by studies that support the use the Veress needle on bariatric patients. In a nationwide survey from the Scandinavian Obesity Surgery registry, the Veress needle was used at the start of 17,445 bariatric operations and an increase in the use of the Veress needle was noticed during the course of the study^19^. Besides, Kosuta et al. concluded that the Veress needle is easier and also relatively safe in this group of obese patients^4^. Interestingly, the data from a related question: “Do you agree that an increase in BMI is directly related to an increased risk of complications during first entry” shows a more skewed distribution.

The vast majority of respondents were interested in testing a new Veress needle that reduces overshooting. Therefore, the results indicate to what extent surgeons think that the currently available Veress needles are adequate and whether improvements to reduce overshooting are relevant. This aligns with the development of many supportive Veress needle systems that should provide extra safety.

Although more than 50% of the respondents suggest that innovation of technology and processes is important for them, only 15% classify themselves as a real innovator. In that category, the vast majority (51% ) see themselves as an early adopter. This suggests that although there is a wish and interest to innovate, many participants simply do not have the time for it or do not know from where to start. This opens the door for better education or more support for surgeons who like to become more innovative in line with the results of Nakaijami et al.^20^.

Regarding the future design of new Veress needles, the survey data shows that it is important to facilitate a good grip on the needle shaft, as most of the respondents hold the needle instead of the grip. The reason is that the grip is located too far from the abdominal wall as some surgeons attempt to stabilise their hand by touching the abdominal wall with their fingers during insertion. The data also indicates that the maximum overshoot should be limited to 0–10 mm. The high response rate and interest in new Veress needle technology that can reduce overshoot does not fully align with the reported incidences Veress needle injuries of e.g. 0.31%^6^ and 0.23%^16^. However, Compeau et al. reported that 57.3% of respondents had either experienced or witnessed a serious laparoscopic entry complication^21^. Therefore we should consider the possibility that the real incidence of such injuries can be much higher than what is reported in the literature.

Regarding the correlation analysis it was expected to see a relation between age and interest in new technology, like the VeressPLUS safety mechanism, or care for new sustainable processes. However, the data does not show this correlation. This suggests that the surgeons who answered this survey have an ongoing interest in sustainable processes and using new innovations that do not diminish when becoming older. Nevertheless, it should be mentioned that the population of surgeons who did not respond out of disinterest could lead to different results, had they answered the questionnaire.

### Limitations

The Executive Office only sent out a reminder to all EAES members to fill out the survey once. As 10% response rate is not very high, it could be useful to send out more reminders to obtain more data. The questionnaire was only validated in a subjective way by sending it out to the four surgeons who are involved in this study. Although the data does not show that some questions were confusing, it could be beneficial to validate this kind of questionnaire with more surgeons with different experience levels and sub-specialty, hailing from different nationalities. It could be that confounders influence the relations between some of the more dichotomous or categorical questions. This should be considered during the interpretation of the weaker relations. Within this survey, potential training or delivery barriers that hamper the integration of Veress needles in daily practice were not considered. Therefore it could be very well that surgeons who wish to use the Veress in daily practice have no access to it. This could have influenced the data obtained. As only EAES members were included in this study, there is a possible selection bias as it is known that gynecologists use the Veress needle more often than surgeons. In follow up studies, this risk should be mitigated by adding associates from other surgical disciplines and geographical locations to the study. This should prevent that survey results represent the EAES group whilst being too conservative or not representative for the total minimally invasive surgical community.

## Conclusion

Safe establishment of pneumoperitoneum largely depends on the technique used to penetrate the abdominal wall. Overshooting of the Veress needle poses a risk for the patient and reduction of overshooting is therefore essential, according to the responding EAES members. The obtained insights and data should be considered during the development of new needle technology for first access devices like the Veress needle.

### Supplementary Information

Supplementary Information 1.

Supplementary Information 2.

Supplementary Information 3.

## Data Availability

All data generated or analysed during this study are included in this publication as supplemental file 1 and 3.
