# Supplementary material for: The relevance of reducing Veress needle overshooting
Source: Sci Rep. 2023 Oct 14;13:17471. doi: 10.1038/s41598-023-44890-1 (PMC10576755; doi:10.1038/s41598-023-44890-1)
Supplement: Supplementary file 1 — Supplementary Information 1. [file 41598_2023_44890_MOESM1_ESM.pdf]

Open vs Closed access

*Vereist

1. What is your preferred method for establishing a pneumoperitoneum in patients with a normal Body Mass Index (BMI)? *

   Markeer slechts één ovaal.

   - Open technique (Hasson method)
   - Closed Technique (Veress Needle)
   - Other

2. What is your preferred method for establishing a pneumoperitoneum in patients with a High BMI? *

   Markeer slechts één ovaal.

   - Open technique (Hasson method)
   - Closed Technique (Veress Needle)
   - Other

3. Roughly, after how many procedures were you comfortable enough in using the Veress needle? *

   ____

4. Roughly, after how many procedures were you comfortable enough in using the Hasson method? *

   ____

5. When did you pick your preferred method? *

Markeer slechts één ovaal.

- During training
- Following personal experience during surgery
- Other

6. Where did you learn how to perform a pneumoperitoneum? * *

Markeer slechts één ovaal.

- In a clinical skills lab
- On the job training
- Both

Preferred access point

Access points

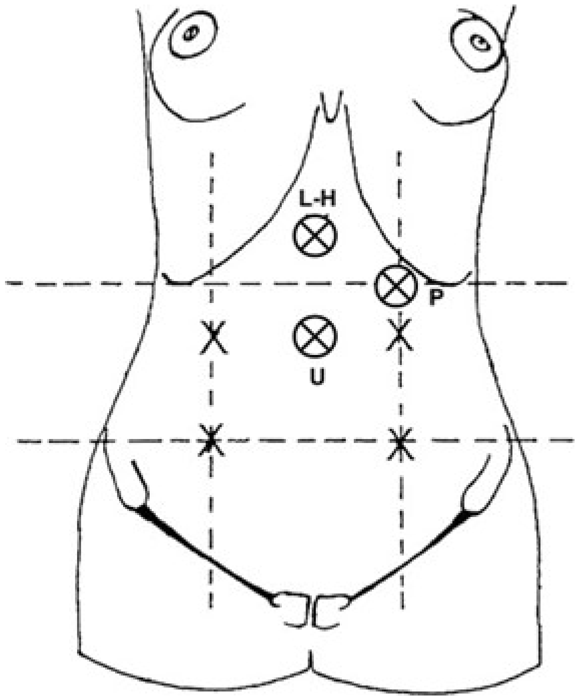

7. What is your preferred (primary) entry location in Normal BMI patients? * *

Markeer slechts één ovaal.

- Lee-Huang point
- Palmer's point
- Umbilical point

8. What is your preferred (primary) entry location in High BMI patients? * *

Markeer slechts één ovaal.

- Lee-Huang point
- Palmer's point
- Umbilical point

Preferred grip

9. How do you hold the Veress needle (see images below)? * *

Markeer slechts één ovaal.

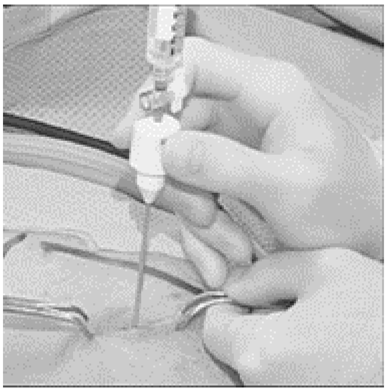

- At the shaft

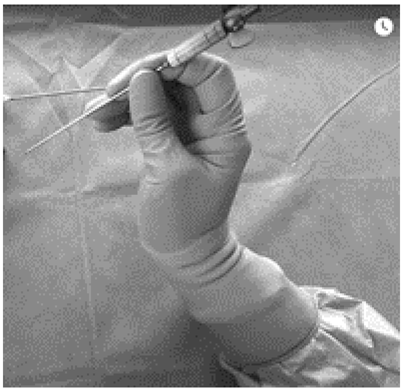

- At the base

10. Do you have a preferred type of Veress needle? *

Markeer slechts één ovaal.

- Yes
- No

11. If yes, which one and why do you prefer it?

Do you agree with the following statements?

12. Decreasing overshooting increases the overall safety of the Closed technique *

Markeer slechts één ovaal.

	1	2	3	4	5	
Strongly disagree						Strongly agree

13. Decreasing the learning curve of the Veress Needle use increases the overall safety of the Closed technique *

Markeer slechts één ovaal.

	1	2	3	4	5	
Strongly disagree						Strongly agree

14. Increase in Body Mass Index is directly related to an increased risk of complications during first entry *

Markeer slechts één ovaal.

	1	2	3	4	5	
Strongly disagree	⬭	⬭	⬭	⬭	⬭	Strongly agree

15. Improving the sustainability of hospital processes and operations is important to me *

Markeer slechts één ovaal.

	1	2	3	4	5	
Strongly disagree	⬭	⬭	⬭	⬭	⬭	Strongly agree

Ideal Insertion Depth

The Ideal Insertion Depth is an acceptable range where the Veress needle tip is confidently within the peritoneal cavity to ensure a safe and efficient insufflation. It is acknowledged that the Ideal Insertion Depth is not a risk free zone. It is a balance between confident entry for safe insufflation while keeping the risk of damaging underlying structures to a minimum.

Overshooting is the point where the Veress needle tip is inserted beyond the necessary level for safe and efficient insufflation

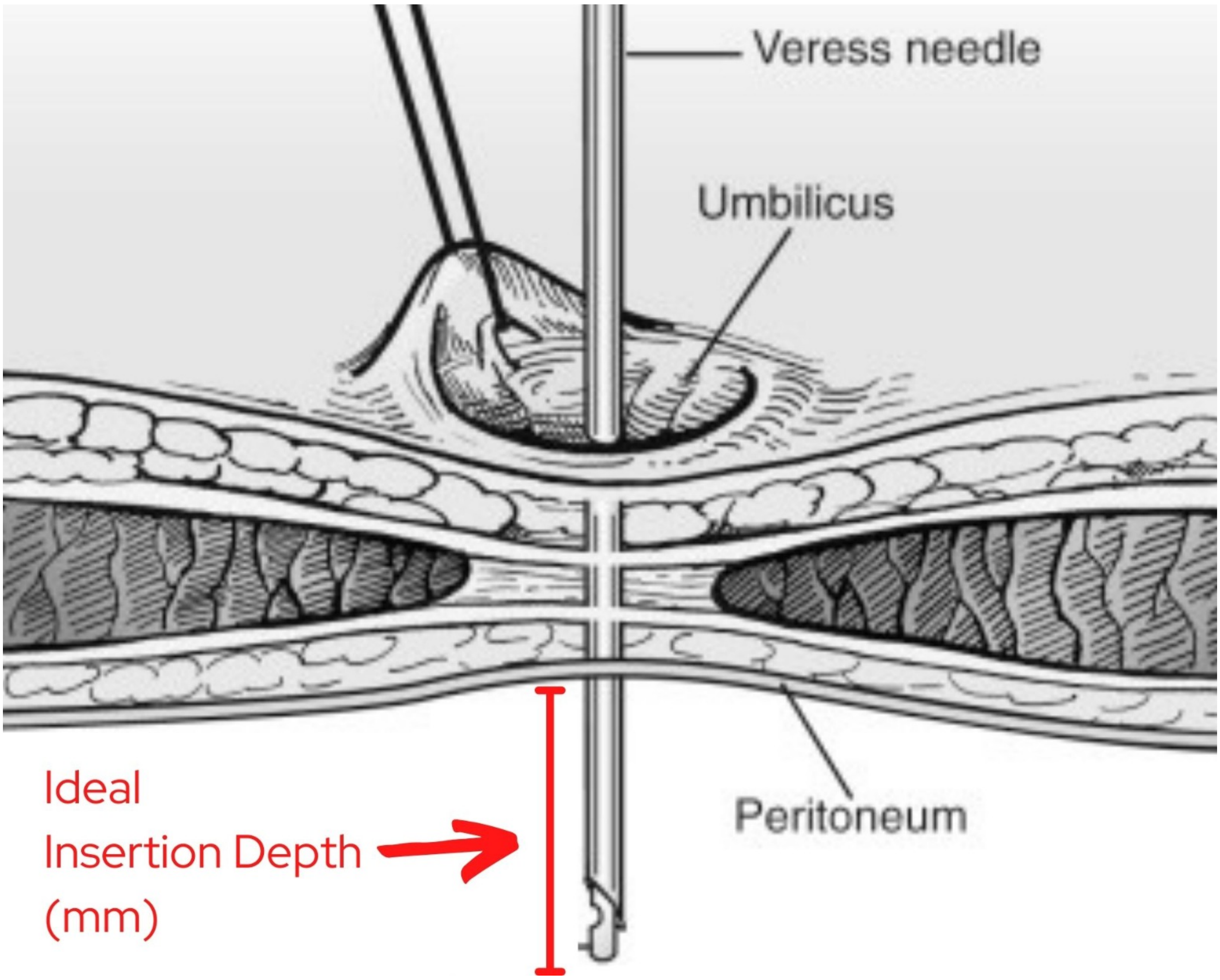
Veress needle
Umbilicus
Peritoneum
Ideal
Insertion Depth
(mm)

Needle tip with ruler (mm increments)

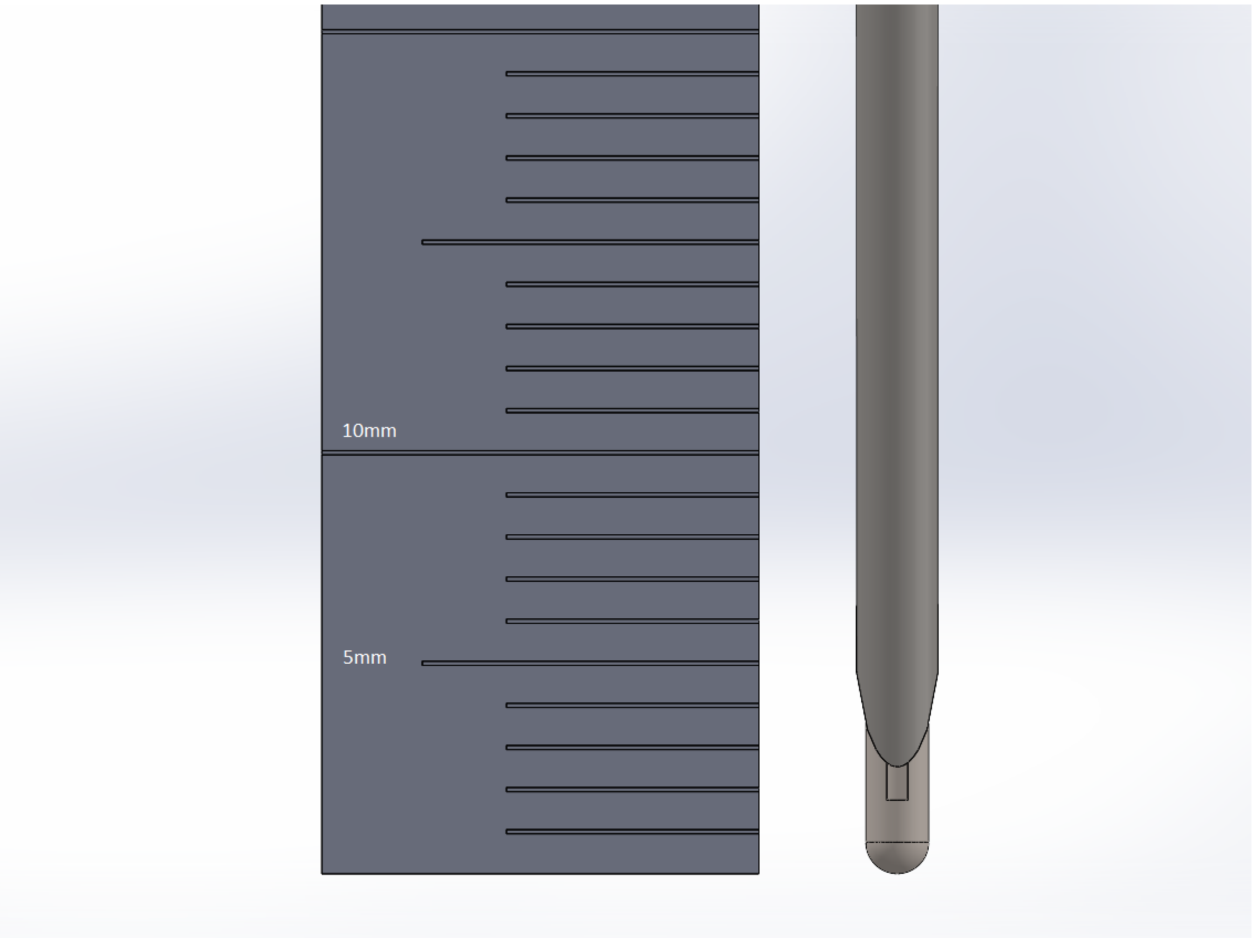

16. In your opinion, how far beyond the peritoneum (Ideal Insertion Depth) does the Veress needle tip need to be for safe insufflation (in mm) * *

New technology

17. In adopting new surgical technology/products do you consider yourself an: * *

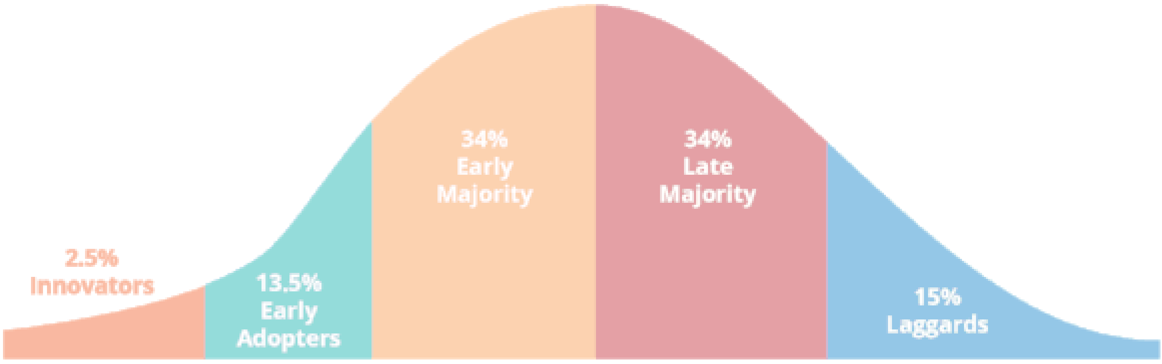

Markeer slechts één ovaal.

- Innovator
- Early adopter
- Early majority
- Late majority
- Laggard

18. What are your main concerns about using NEW medtech devices during surgery? * *

Veress PLUS	The Veress PLUS is a novel veress needle with an addon safety device that reduces overshooting by 60 - 80%. The Veress PLUS is a research collaboration between ProVinci Medtech, Van Straten Medical, Technical University of Delft, Amsterdam University Medical Center, The University of Malta and the Heidelberg University.<br><br>Features of the Veress PLUS<br>1. Reduces overshooting by 60-80%<br>2. Reduces the learning curve among new surgeons. In preclinical studies new surgeons consistently performed like experienced surgeons in under 5 attempts<br>3. The Veress PLUS looks and feels like the traditional veress needle and therefore requires no change in technique among experienced surgeons<br>4. Sustainable

19. If the above claims are true would you consider using the new Veress PLUS in patients with a normal BMI? * *

Markeer slechts één ovaal.

	1	2	3	4	5	
No	⬭	⬭	⬭	⬭	⬭	Yes

20. Please explain your answer *

____

21. If the above claims are true would you consider using the new Veress PLUS in patients with a HIGH BMI? * *

Markeer slechts één ovaal.

	1	2	3	4	5	
No	⬭	⬭	⬭	⬭	⬭	Yes

22. Please explain your answer *

____

Demographic data

23. Age *

____

24. Gender *

Markeer slechts één ovaal.

- Male
- Female
- Prefer not to say
- Anders: ____________________

25. Nationality *

____

26. Years of experience (as a surgeon) *

____

27. Estimated number of Veress needle use *

____

28. Place of work *

Markeer slechts één ovaal.

- Training hospital
- Non-training hospital
- Private clinic
- Academia
- Other

29. Surgical specialty *

____

30. Dominant hand *

Markeer slechts één ovaal.

- Left
- Right
- Ambidextrous

31. Would you be interested in joining the Veress PLUS R&D project as an innovator/early adopter? *

Markeer slechts één ovaal.

- Yes
- No
- Maybe

32. If yes, please share your contact details below (or email us on d.cefai@provinci-medtech.com):(Name/Surname/Title/Email)

____

Deze content is niet gemaakt of goedgekeurd door Google.

Google Formulieren
