# Supplementary material for: The relevance of reducing Veress needle overshooting
Source: Sci Rep. 2023 Oct 14;13:17471. doi: 10.1038/s41598-023-44890-1 (PMC10576755; doi:10.1038/s41598-023-44890-1)
Supplement: Supplementary file 2 — Supplementary Information 2. [file 41598_2023_44890_MOESM2_ESM.pdf]

Consent Form

Title of Study: **The relevance of Veress needle overshooting reduction**

Investigator(s): Roelf Postema, David Cefai, Tim Horeman-Franse

Introduction:
You are invited to participate in a questionnaire survey aimed at exploring the experiences and perceptions of surgeons with regards to the relevance of Veress needle overshooting reduction. Your participation in this survey is voluntary, and your responses will remain anonymous.

Procedure:
Participation in this study will involve completing an online questionnaire, which will take approximately 20 minutes to complete. The questionnaire will consist of 32 questions, and you will be asked to provide responses to each question to the best of your ability.

Benefits:
There are no direct benefits to you for participating in this study. However, your participation may contribute to a better understanding of the relevance of Veress needle overshooting, which may have potential benefits for future surgical practice and patient outcomes.

Risks:
There are no known risks associated with participating in this study.

Confidentiality:
All data collected in this study will be kept strictly confidential. All data will be kept in a secure location and will only be accessible to the investigators. Depersonalised data well be used in for scientific publication

Voluntary Participation:
Participation in this study is entirely voluntary. You have the right to withdraw from the study at any time without penalty or loss of benefits. Your decision to participate or not will not affect your current or future relationship with the investigators or the European Association of Endoscopic Surgeries.

Contact Information:
If you have any questions or concerns regarding this study, you may contact the investigators at d.cefai@provinci-medtech.com.

Consent:
By completing and submitting the questionnaire, you are indicating that you have read and understood the information provided in this consent form and agree to participate in this study.

Thank you for your participation in this study.
